# Supplementary material for: Stroke among cancer patients
Source: Nat Commun. 2019 Nov 15;10:5172. doi: 10.1038/s41467-019-13120-6 (PMC6858303; doi:10.1038/s41467-019-13120-6)
Supplement: Supplementary file 1 — Supplementary Information [file 41467_2019_13120_MOESM1_ESM.pdf]

SUPPLEMENTARY NOTES

Calendar period, registry, and diversity

The SEER program has evolved since its inception in the United States in 1973.[1] As of 2017, SEER has up to 36 years of longitudinal and ongoing data collection, with a representative sample size of more than 6 million cancer cases, and a comprehensive quality assurance process. Over time, more registries were added to SEER; in the current analysis, the SEER 18 (adjusted for Hurricane Katrina Impacted Louisiana cases) and SEER 9 registries were used. The registry number denotes the number of registries. *SEER 9*. The first areas included at that time were Connecticut, Hawaii, Iowa, San Francisco/Oakland, and Detroit. Geographic areas were included based on two objectives: (1) the ability of a geographic cancer registry to maintain high-quality data (explained below), and (2) having a population that represents minority subpopulations.[1]

In 1974-1975, the metropolitan areas of Atlanta and Seattle/Puget Sound were added, and the SEER 9 registry was finalized. *SEER 11*. In order to expand on the second objective, two more registries were added, Los Angeles County and 4 Counties in the San Jose/Monterey area. These counties included cases diagnosed after 1992. *SEER 13*. The next grouping additionally included 10 predominantly African American counties of rural Georgia and the Alaska Native American Tumor Registry. *SEER 17*. For cancers diagnoses after 2001, four additional areas were included: the remaining counties of California, Kentucky, Louisiana, and New Jersey. These counties have supplemental funding by the Centers for Disease Control (CDC). Based on the inclusion of these areas, the SEER database is representative of the population of the USA, and this has been validated by external studies.[1]

Since the SEER database have increased the proportion of the US population captured over the years, in early years of the SEER program there are fewer survivors than in later years, and the proportion of death by index cancer is lower in later years. Further, the rate count of people having a cancer depends on the number of patients living with this cancer from previous years (which depends on cancer prevalence), those diagnosed within the calendar year (which depends on screening and incidence), and those dying during that year (which depends on cancer and treatment aggressiveness, how death is coded, common risk factors among cancers and comorbidities, and patient age). Certain cancers have an indolent course (e.g. prostate), and patients diagnosed in subsequent years are added to the cumulative count, increasing the number of prostate cancer patients relative to all others; for patients with aggressive cancers (e.g. pancreatic), the addition of patients diagnosed in subsequent years has little effect on the cumulative number because of high rates of mortality.

Age

SEER provides age-standard adult (age ≥15) cancer populations to calculate age-standardized survival, which is used to compare survival across time or different cancer populations with different age distributions. The standards provided are the International Cancer Survival Standard (ICSS) derived for three broad groups of cancer sites with similar patterns of incidence by age. ICSS 1 includes cancer sites with increasing incidence by age (most cancer sites; e.g. prostate). ICSS 2 includes cancer sites with broadly constant incidence by age (e.g. nasopharynx). ICSS 3 includes cancer sites that mainly affect young adults (e.g. testis). By using the appropriate standard, the age-standardized survival is theorized to be like the raw (un-weighted) survival. For each of the three ICSS populations, SEER*Stat provides weights by 5-year age bins using the age variable, Age recode with <1 year olds, and by five larger age

groups, in the variable, Age Standard for Survival (15-44, 45-54, 55-64, 65-74, 75+), as described on the SEER website.

Quality assurance and completeness

SEER undergoes quality assurance using systematic, standardized, and periodic data collection procedure for all defined members of a defined cohort is performed to avoid surveillance bias.[1] The case-finding audits are performed by a qualified member from each SEER registry under the direction of members of the National Cancer Institute. Auditors create an abstract the contains the primary site and the case finding source.[2] When performing audits, SEER adheres to two basic principles: auditing high quantity and high risk data. High quantity refers to disease sites that have the highest incidence and prevalence (e.g. breast, prostate, lung, colon); as well facilities that contribute the greatest percent of cases to the central database. Additionally, pathology laboratories are selected to review tissue from patients not seen at that hospital. High risk refers to cases that are likely to be miscoded (e.g. head and neck, hematopoietic diseases); compliance to new rules; and newly-reportable diseases.

Defining the cause of death

Mortality codes in SEER are assigned from death certificates, completed by the doctor caring for the patient at the time of demise. There is no single best method for calculating survival from cancer in the SEER program.[3] Different methods can give different outcomes, but for most variants considered the differences are small. For stroke, there is likely little discrepancy in the cause of death, as compared to a cause of death like heart disease, which may be cause by the cancer treatment, underlying heart disease, or a combination of both.

Data session information

The instructions to access the SEER data are provided below:

(1) Download the SEER*Stat software from the NCI website: https://seer.cancer.gov/seerstat/software/

(2) Open the program

(3) Click “File”, “New,”

“MP-SIR” Session to generate the SMRs. Note, this was used in Table 1 and Figure 1 of the current analysis.

“Case Listing” to generate a list of patient cases diagnosed.

“Incidence” to generate a list of the incidence of cancer or cause of death.

Note, this was used in Table 2 and Figure 2 of the current analysis, and to generate the ORs.

(4) Click on the desired registry to use for each of the sessions. For the purposes of this analysis, the following registries and options were selected.   All the other data supporting the findings of this study are available within the article and its supplementary information files and from the corresponding author upon reasonable request

In the “MP-SIR” session, select the following:

The output of these sessions is provided in Source Data file.

Intricacies of Surveillance, Epidemiology, and End Results (SEER) Databases

Registry Differences

The SEER databases have been evolving over the years, and this evolution is described in our Data Availability Statement and by previous work.[1,4-6] Briefly, SEER covers key demographic areas in the United States, and these areas/databases have slowly been added to SEER since the 1970s. The SEER 9 database includes 9 registries from 1973-2014; the SEER 18

database contains 18 registries, including the most recent patients from 2000-2016. Notably, SEER 18 is not limited to this time period; rather, the "2000-2016" refers to when all databases are collecting the data. Prior databases and their patients (before 2000) are available in SEER 18. The SEER 21 database was released in 2019, including more geographic regions. As data are collected from more regions, the same concepts of patient inclusion over time apply.

SEER is able to analyze data by different methods, using its "Sessions." The time period of these data sessions depend on the SEER database chosen (SEER 9, SEER 18, SEER 21, etc.). The "Standardized Incidence Ratio (SIR) session" provides incidence of a particular event after diagnosis, as a function of follow up time or age at diagnosis. When the event of interest is death as a function of follow up time, the SIRs are actually standardized mortality ratios (SMRs), and they provide the relative risk of death from a particular cause vs. the general population.

A "case listing" session is another option in displaying the data. Case listing sessions provide patient-level data, with each patient in a row, and variables (e.g. age, sex, cancer type) in columns. Thus, case listing sessions may be used to calculate odds ratios and generate survival plots.

Calculating Standardized Mortality Ratios

SMRs consist of two measures: (observed number of events, during time at risk) / (expected number of events in the reference population, during time at risk). SMRs may be calculated as a function of different times at risk, including time after diagnosis (i.e. the latency period) or age at diagnosis. When SMRs are calculated as a function of time after diagnosis, they provide the relative risk of death from one particular cause vs. the reference population. The reference population changes depending on the population and the time period. Thus, SMRs should not be compared to one another, and they would be expected to vary over different time

periods or with different patient populations. Further, calculated SMRs may differ when using different SEER databases because (1) the observed number of events of interest among cancer patients may change, and (2) the number of events of interest in the reference population (i.e. the United States) also changes over the years.

Latency Exclusion Periods in Standardized Mortality Ratios

For SMRs calculated as a function of follow up time, SMRs during each window of time (e.g. at 1 year after diagnosis, 1-5 years after diagnosis, etc.) depend on the time at risk. With longer time at risk and more observed events, the confidence intervals become smaller, and measurements are more accurate. With a short time at risk (e.g. the first few months after diagnosis), or very few events (e.g. suicide), or among a niche patient cohort (e.g. Hodgkin lymphoma), the confidence intervals can widen dramatically.

In the first few months after diagnosis of cancer, patients often have an "introduction to the medical system;" i.e. a patient living in a rural area comes to a hospital where they are diagnosed with cancer, as well as many other comorbidities like heart disease, lung dysfunction, kidney failure, etc. The patient may die of any of these within a few months, but estimating the observed versus expected rate of death becomes difficult, and the confidence intervals for an SMR naturally widen. Thus, some researchers, including our team, sometimes elect to exclude the first 2 months from the SMR calculations. While SMRs may actually be very high during this time, the confidence intervals are so wide that an accurate measure is not meaningful. Moreover, the absolute number of observed events in this time may be rather low, especially when the event of interest is rare. Thus, the overall SMRs for the entire follow up period (with or without the latency periods) tend to be relatively similar.

REFERENCES

1 Park, H. S., Lloyd, S., Decker, R. H., Wilson, L. D. & Yu, J. B. Overview of the Surveillance, Epidemiology, and End Results database: evolution, data variables, and quality assurance. *Curr. Probl. Cancer* **36**, 183-190, doi:10.1016/j.currproblcancer.2012.03.007 (2012).

2 *National Cancer Institute. Casefinding Studies - SEER Quality Improvement.*, <http://seer.cancer.gov/qi/tools/casefinding.html> (2016).

3 Boer, R. *et al.* (Statistical Research and Applications Branch, NCI, Bethesda, MD).

4 Park, H. S., Lloyd, S., Decker, R. H., Wilson, L. D. & Yu, J. B. Limitations and biases of the Surveillance, Epidemiology, and End Results database. *Curr. Probl. Cancer* **36**, 216-224, doi:10.1016/j.currproblcancer.2012.03.011 (2012).

5 Zaorsky, N. G. *et al.* Suicide among cancer patients. *Nature communications* **10**, 207, doi:10.1038/s41467-018-08170-1 (2019).

6 Zaorsky, N. G. *et al.* Causes of death among cancer patients. *Ann. Oncol.* **28**, 400-407, doi:10.1093/annonc/mdw604 (2017).

SUPPLEMENTARY TABLES

Supplementary Table 1. SMRs and 95% CI for pediatric cancer patients who died of stroke.

SMR (95% CI)	<1 year	1-5 years	5-10 years	10+ years	Total Person Years at Risk
All Sites	240.55 (49.61-703)	154.45 (56.68-336.16)	125.15 (25.81-365.74)	210.16 (77.13-457.44)	138.82
Digestive System	0 (0-20396.48)	1393.32 (35.28-7763.11)	0 (0-0)	0 (0-0)	4.25
Stomach	0 (0-20396.48)	1393.32 (35.28-7763.11)	0 (0-0)	0 (0-0)	4.25
Urinary System	0 (0-1369.26)	126.89 (3.21-706.98)	18991.13 (480.81-105811.79)	0 (0-0)	7
Kidney and Renal Pelvis	0 (0-1369.26)	126.89 (3.21-706.98)	18991.13 (480.81-105811.79)	0 (0-0)	7
Eye and Orbit	682.24 (17.27-3801.21)	0 (0-0)	0 (0-0)	0 (0-0)	0.67
Brain and Other Nervous System	0 (0-1472.46)	113.41 (2.87-631.89)	67.42 (1.71-375.66)	199.19 (54.27-510)	79.09
Brain	0 (0-1472.46)	113.41 (2.87-631.89)	67.42 (1.71-375.66)	199.19 (54.27-510)	79.09
Endocrine System	0 (0-2342.34)	0 (0-833.54)	429.17 (10.87-2391.16)	130.12 (3.29-725)	27.32
Other Endocrine including Thymus	0 (0-2342.34)	0 (0-833.54)	429.17 (10.87-2391.16)	130.12 (3.29-725)	27.32
Lymphoma	644.44 (16.32-3590.56)	104.22 (2.64-580.69)	0 (0-0)	0 (0-0)	4.67
Hodgkin Lymphoma	0 (0-2528.35)	104.22 (2.64-580.69)	0 (0-0)	0 (0-0)	4.58
Hodgkin - Nodal	0 (0-2528.35)	104.22 (2.64-580.69)	0 (0-0)	0 (0-0)	4.58
Non-Hodgkin Lymphoma	10782.92 (273-60078.61)	0 (0-0)	0 (0-0)	0 (0-0)	0.08
NHL - Nodal	10782.92 (273-60078.61)	0 (0-0)	0 (0-0)	0 (0-0)	0.08
Leukemia	400.2 (10.13-2229.79)	269.83 (32.68-974.71)	0 (0-545.91)	1277.61 (32.35-7118.38)	15.82
Lymphocytic Leukemia	0 (0-1523.04)	269.83 (32.68-974.71)	0 (0-545.91)	1277.61 (32.35-7118.38)	15.49
Acute Lymphocytic Leukemia	0 (0-1523.04)	269.83 (32.68-974.71)	0 (0-545.91)	1277.61 (32.35-7118.38)	15.49
Myeloid and Monocytic Leukemia	13041.81 (330.19-72664.31)	0 (0-0)	0 (0-0)	0 (0-0)	0.34
Acute Myeloid Leukemia	13041.81 (330.19-72664.31)	0 (0-0)	0 (0-0)	0 (0-0)	0.34

SUPPLEMENTARY FIGURES

Supplementary Figure 1. Standardized mortality ratios (SMRs) of fatal stroke in head and neck cancer patients by subsite.

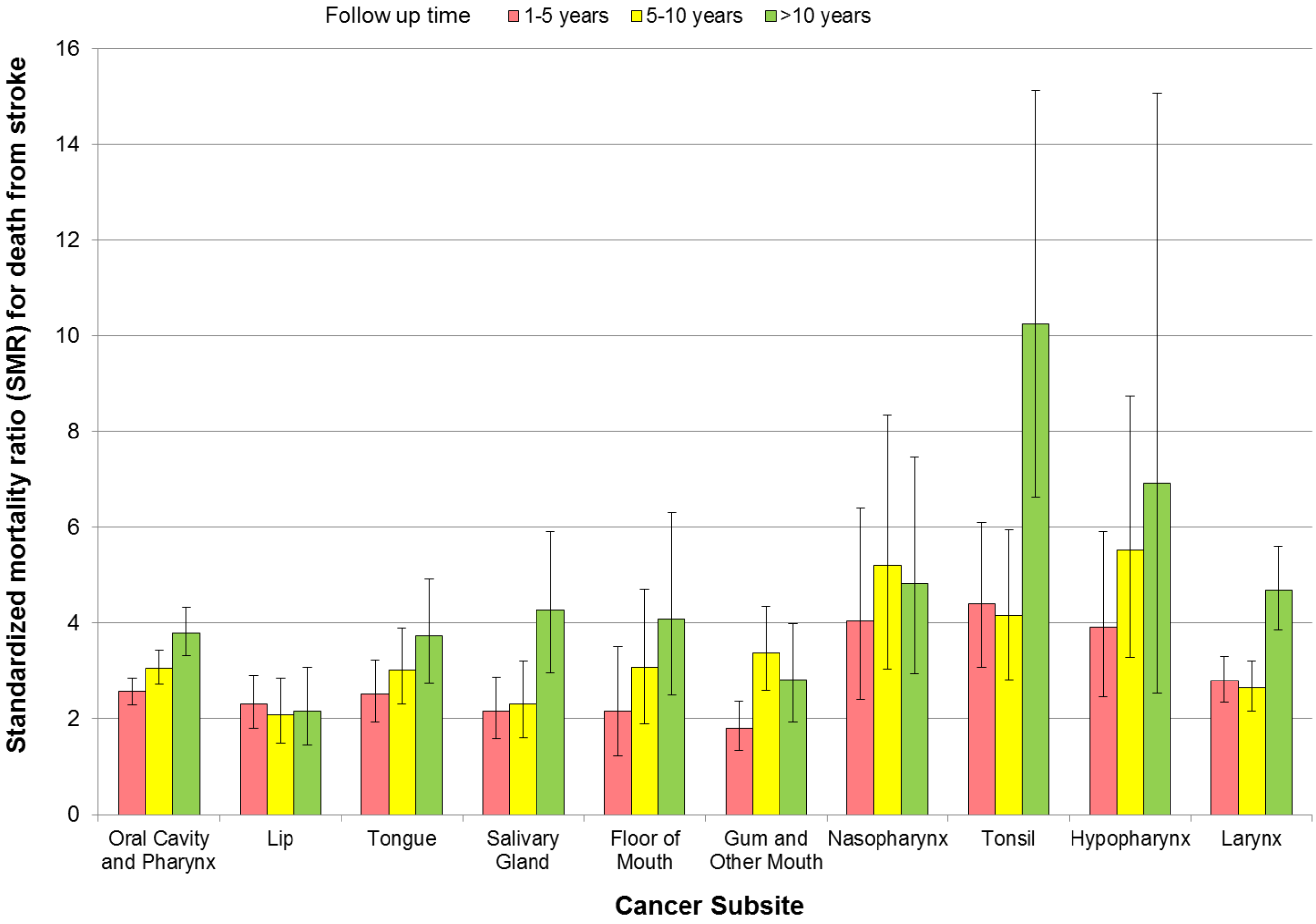

DATA SET LEGENDS

Source Data 1. The following SEER data base was used to collect this data: Incidence - SEER 18 Regs Research Data + Hurricane Katrina Impacted Louisiana Cases, Nov 2017 Sub (1973-2015 varying) - Linked To County Attributes - Total U.S., 1969-2016 Counties. The data provided is the case listing file for all patients who died of stroke from the years 1992 to 2015. Relevant SEER session information is provided. This data was used in Tables 1 and 2.

Source Data 2. The following SEER data base was used to collect this data: Incidence - SEER 9 Regs Research Data, Nov 2017 Sub (1973-2015) <Katrina/Rita Population Adjustment> - Linked To County Attributes - Total U.S., 1969-2016 Counties. The data provided is a rate session for all patients who died of stroke from 1973 to 2015. Relevant SEER session information is provided. This data was used in Table 1.

Source Data 3. The following SEER data base was used to collect this data: Incidence - SEER 13 Regs excluding AK Research Data, Nov 2017 Sub (1992-2015) for SMRs - Linked To County Attributes - Total U.S., 1969-2016 Counties. The data provided is the standardized mortality ratios and corresponding 95% confidence intervals for all patients who died of stroke, sorted by months since diagnosis. Relevant SEER session information is provided. This data was used in Figure 1.

Source Data 4. The following SEER data base was used to collect this data: Incidence - SEER 13 Regs excluding AK Research Data, Nov 2017 Sub (1992-2015) for SMRs - Linked To County Attributes - Total U.S., 1969-2016 Counties. The data provided is the standardized

mortality ratios and corresponding 95% confidence intervals for all patients who died of stroke, sorted by age at diagnosis. Relevant SEER session information is provided. This data was used in Figure 2.

Source Data 5. The following SEER data based was used to collect this data: Incidence - SEER 9 Regs Research Data, Nov 2017 Sub (1973-2015) <Katrina/Rita Population Adjustment> - Linked To County Attributes - Total U.S., 1969-2016 Counties. The data provided is rate of death from stroke, sorted by age at diagnosis. This data was used in Figure 3.

Supplementary Data 1. The following SEER data base was used to collect this data: Database: Incidence - SEER 13 Regs excluding AK Research Data, Nov 2017 Sub (1992-2015) for SMRs - Linked To County Attributes - Total U.S., 1969-2016 Counties. The data provided is the standardized mortality ratios and corresponding 95% confidence intervals for pediatric patients diagnosed from 1992-2015 who died of stroke. Relevant SEER session information is provided.

Supplementary Data 2. The following SEER data base was used to collect this data: Database: Incidence - SEER 13 Regs excluding AK Research Data, Nov 2017 Sub (1992-2015) for SMRs - Linked To County Attributes - Total U.S., 1969-2016 Counties. The data provided is the standardized mortality ratios and corresponding 95% confidence intervals for patients diagnosed with head and neck cancers from 1992-2015 who died of stroke. Relevant SEER session information is provided.

Supplementary Data 3. The following SEER data base was used to collect this data: Database: Incidence - SEER 13 Regs excluding AK Research Data, Nov 2017 Sub (1992-2015) for SMRs - Linked To County Attributes - Total U.S., 1969-2016 Counties. The data provided is the standardized mortality ratios and corresponding 95% confidence intervals for patients diagnosed from 1992-2002 who died of stroke, and patients diagnosed from 2005-2015 who died of stroke. Relevant SEER session information is provided.
