# Supplementary material for: Stroke among cancer patients
Source: Nat Commun. 2019 Nov 15;10:5172. doi: 10.1038/s41467-019-13120-6 (PMC6858303; doi:10.1038/s41467-019-13120-6)
Supplement: Supplementary file 3 — Description of Additional Supplementary Files [file 41467_2019_13120_MOESM3_ESM.docx]

**Description of Supplementary Files**

**File Name: Supplementary Data 1**

**Description:** The following SEER data base was used to collect this data: Database: Incidence - SEER 13 Regs excluding AK Research Data, Nov 2017 Sub (1992-2015) for SMRs - Linked To County Attributes - Total U.S., 1969-2016 Counties. The data provided is the standardized mortality ratios and corresponding 95% confidence intervals for pediatric patients diagnosed from 1992-2015 who died of stroke. Relevant SEER session information is provided.

**File Name: Supplementary Data 2**

**Description:** The following SEER data base was used to collect this data: Database: Incidence - SEER 13 Regs excluding AK Research Data, Nov 2017 Sub (1992-2015) for SMRs - Linked To County Attributes - Total U.S., 1969-2016 Counties. The data provided is the standardized mortality ratios and corresponding 95% confidence intervals for patients diagnosed with head and neck cancers from 1992-2015 who died of stroke. Relevant SEER session information is provided.

**File Name: Supplementary Data 3**

**Description:** The following SEER data base was used to collect this data: Database: Incidence - SEER 13 Regs excluding AK Research Data, Nov 2017 Sub (1992-2015) for SMRs - Linked To County Attributes - Total U.S., 1969-2016 Counties. The data provided is the standardized mortality ratios and corresponding 95% confidence intervals for patients diagnosed from 1992-2002 who died of stroke, and patients diagnosed from 2005-2015 who died of stroke. Relevant SEER session information is provided.
